# Varying Association Between Cancer Caregiver Mental Health and Area Deprivation by Household Income

**DOI:** 10.1093/geroni/igaf122.2730

**Published:** 2025-12-31

**Authors:** Kyle Pitzer, Karla Washington, Darrell Hudson, Keisha White Makinde, Todd Becker, Debra Oliver, Jacquelyn Benson, George Demiris

**Affiliations:** Washington University in St. Louis, St. Louis, Missouri, United States; Washington University in St. Louis, St. Louis, Missouri, United States; University of Michigan, Ann Arbor, Michigan, United States; Washington University in St. Louis, St. Louis, Missouri, United States; WashU Medicine, St. Louis, Missouri, United States; Washington University in St. Louis, St. Louis, Missouri, United States; Washington University in St. Louis, St. Louis, Missouri, United States; University of Pennsylvania, Philadelphia, Pennsylvania, United States

## Abstract

In the United States, nearly 9 in 10 newly diagnosed cancer patients are age 50 and above; almost 6 in 10 are over 65. Consequently, older adults and their family caregivers disproportionately bear the burden of this illness, often leading to pronounced mental health concerns. While individual and interpersonal factors related to cancer caregivers’ well-being have been extensively studied, little is known about how community-level factors are related to their mental health. Using data from a large randomized clinical trial of a caregiver support intervention, this secondary data analysis examined the association between area deprivation and mental health among cancer caregivers as well as differential associations based on household income. To determine area deprivation, participants were geocoded to determine their census block group and corresponding area deprivation index percentile. Linear models were then estimated to assess the association between area deprivation and anxiety and depression while adjusting for several demographic and caregiving context covariates. The interaction between area deprivation and household income was also examined. While there was no significant association between area deprivation and depression regardless of household income, models indicated a cross-over association between area deprivation and anxiety by household income. Specifically, caregivers with annual household incomes below $70,000 had greater anxiety as area deprivation increased while caregivers with household incomes above $70,000 had less anxiety as area deprivation increased, on average. These findings highlight the importance of considering both the financial situation and the living environment of cancer caregivers when developing supportive interventions.

